# A Prescription for Improving Drug Formulary Decision Making

**DOI:** 10.1371/journal.pmed.1001220

**Published:** 2012-05-22

**Authors:** Gordon D. Schiff, William L. Galanter, Jay Duhig, Michael J. Koronkowski, Amy E. Lodolce, Pam Pontikes, John Busker, Daniel Touchette, Surrey Walton, Bruce L. Lambert

**Affiliations:** 1 Brigham and Woman's Hospital, Harvard Medical School, Boston, Massachusetts, United States of America; 2 Center for Education and Research on Therapeutics, University of Illinois at Chicago, Chicago, Illinois, United States of America; 3 University of Illinois at Chicago, College of Medicine, Chicago, Illinois, United States of America; 4 Department of Pharmacy Practice, University of Illinois at Chicago, Chicago, Illinois, United States of America; 5 University of Illinois at Chicago, Department of Pharmacy Administration, Chicago, Illinois, United States of America; 6 John H. Stroger Jr. Hospital of Cook County, Chicago, Illinois, United States of America

## Abstract

Gordon Schiff and colleagues present a new tool and checklist to help formularies make decisions about drug inclusion and to guide rational drug use.

**Summary Points**

- Drug formularies are widely used by hospitals, health systems, and private and national drug insurance plans. Although considerable attention has been devoted to their role in cost containment, formularies' role in guiding rational drug use remains underdeveloped and could be enhanced by a more standardized critical evaluation of drugs proposed for formulary placement.
- We developed a tool based on a project at two US public academic hospitals consisting of a six-domain checklist of questions for evaluating drugs requested for addition.
- The tool poses 48 questions related to: evidence of need, efficacy, medication safety, misuse potential, cost issues, and decision-making process.
- The checklist can facilitate more standardized and critical scrutiny of the evidence and therapeutic alternatives. It can educate new committee members, guide discussions of drugs proposed for formulary addition, and be used to evaluate the quality of committee decision making.
- Limitations include its generalizability to all types of formulary committees and settings, and lack of time and data for committees to fully address all checklist questions.

## Introduction

Drug formularies are a ubiquitous, heterogeneous yet often contentious feature of both US and international drug policy [1]–[6]. Formularies represent the fundamental approach embodied in the World Health Organization (WHO) Model Formulary 2004 and various countries' essential medicines lists [1]. In addition, WHO encourages each hospital to establish a drug and therapeutics committee to oversee selection of drugs and to set policies for that institution's local formulary [7]. Formularies and committees that oversee them are present in some form in virtually every US hospital and outpatient drug plan and are highly visible components of public drug benefits in many countries. Thus decisions made by these committees directly or indirectly impact every prescriber, pharmacist, and patient [8]–[11].

**Figure 1:**
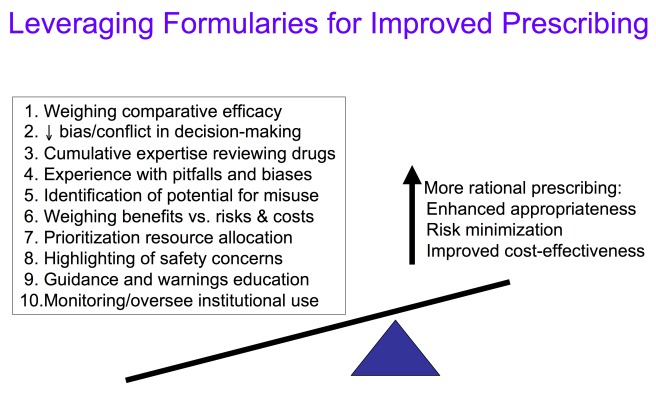
Leveraging formularies for improved prescribing. Formularies are poised to enhance the quality of drug prescribing decision making beyond that of individual practitioners. By encoding the collective expertise and judgment of a group of physicians and pharmacists who have resources and experience to weigh evidence in context, formularies work in ten ways to help optimize prescribing. In addition to supporting the decision making of individual clinicians, the formulary committee oversees the safe and effective use of drugs in institutions by providing guidance and engaging in ongoing drug utilization review.

While some clinicians criticize formularies for limiting clinical autonomy, others have argued that formularies have strayed from their original mission—to identify and designate drugs of choice to guide more rational prescribing—claiming instead they have become overly focused on cost containment and created needless hurdles and complexities for physicians and patients [10],[12],[13]. Nonetheless, formularies can unquestionably exert a powerful influence on prescribing decisions and medication utilization [14]–[19]. At their best, as vehicles and venues for identifying, weighing, and designating best evidence, formularies can assess, teach, and guide prescribing toward the most appropriate and evidence-based choices, helping to direct use toward the most efficacious, safest, and cost-effective therapies, while serving as a firewall to protect against prescribing overly driven by marketing claims [11]–[13],[16]–[21]. Through the decision-making activities of the formulary process, knowledge and leverage may be applied to enhance prescribing practices and patient outcomes in ways that go beyond initial regulatory approval and individual prescribers' ability to weigh the role and value of new medications (Figure 1).

## Tool Development

As members and chairs of multiple US formulary committees, we observed that the depth and quality of debate, discussion, and deliberation around decisions to add a product to the formulary varied tremendously from drug to drug, committee to committee, and meeting to meeting. Lacking a more rigorous framework for evaluating requested drugs, and faced with packed meeting agendas that never seemed to permit sufficient time to thoroughly explore decision pros and cons, monitoring requirements, and optimal usage for proposed new drugs, we observed that discussions were often subjective, unsystematic, and incomplete [13].

As part of a project sponsored by the US Attorney General Consumer and Prescriber Education Grant Program (funded by the Neurontin settlement [22]), we had the opportunity to bring together formulary committee chairs and participants from two public teaching hospitals and a university-based school of pharmacy. Our initiative, entitled the Formulary Leveraged Improved Prescribing (FLIP) Project was continued through the work of a US Agency for Healthcare Research and Quality–funded Center for Education and Research in Therapeutics [23].

On the basis of our earlier research [13] and more recent survey data from the Institute for Safe Medication Practices (ISMP) [24],[25], we concluded that there was a need to overcome various misconceptions about formularies. We saw an opportunity to redirect formulary committee discussions away from less pertinent issues (often reflecting differing general assumptions and personal biases about the fundamental purpose and role of formularies) and toward more deliberative evaluation of the evidence related to the particular drugs and indications under review.

To address this need, we developed and refined structured criteria to help direct this critical evaluative process. We developed a checklist on the basis of the experiences of the two formulary committee chairs (GDS, WLG) along with our FLIP team members, which included two general internists (pharmacy and therapeutics [P&T] Chairs), five pharmacists, a former pharmaceutical sales representative, two pharmacoeconomists, and a pharmacy communication expert. We began by compiling a list of frequent questions and issues arising in deliberations of the two P&T Committees. The list was then grouped into six domains and iteratively refined by evaluating its comprehensibility, usefulness, completeness, and applicability to formulary committee meetings over the course of the 2-year project. Additional input (and eventual incorporation of selected portions of the checklist) was obtained from the University of Illinois at Chicago (UIC) Drug Information Group and the Academy of Managed Care Pharmacy (AMCP), both with extensive experience preparing drug monographs used for formulary committee decision making.

## Formulary Drug Application Evaluation Tool

The FLIP formulary drug application evaluation tool poses a series of questions that provide a framework for formulary decision making (Box 1). These questions are designed to assist formulary committee members in evaluating claims made about drugs being considered for addition to the formulary and, if added, to assist in deciding what restrictions or special monitoring precautions should be put in place. Emphasis centers on the quality of the available evidence and on comparisons to therapeutic alternatives. The ultimate objective of the tool is to help committee members critically evaluate the role for a given drug in significantly improving patient outcomes related to specific indication(s).

**Box 1. Checklist Tool for Guiding Formulary Decision Making**

**A. Evidence of need**

***Is there a compelling need to add the drug to our formulary?***

- What is the prevalence and importance of the condition the drug is intended to treat? What is the relevance of this drug to our population? Are there special subpopulations for which there may be a compelling need?
- What are the demonstrated shortcomings of existing therapy? Is there evidence that this drug overcomes problems in safety, efficacy, acceptability, or convenience that characterize existing therapy?
- What role does this drug play in addressing this need? What are the US Food and Drug Administration (FDA), European Medicines Agency (EMA), or other international agencies' approved indications? What other claims for the drug are being made?
- What other therapeutic approaches (including non-drug alternatives) might reasonably be pursued instead?
- Is the drug needed for all the venues/settings for which it is being requested (e.g., for both inpatient and outpatient formulary use)?

**B. Efficacy**

***What is the evidence to support the claims for this drug?***

- What is the quality and strength of the evidence supporting the efficacy claims? How well designed are these studies?
- Are the claims (both on- and off-label) being made for this drug supported by the data presented?
- How relevant is the population in the published studies to our population and patients in whom it is likely to be used? Were patients like those we treat included in the clinical trials used to gain FDA, EMA, or other governmental regulatory approval, and will the drug's use likely be similar to patients where benefit is proven?
- To what extent are the benefits based on surrogate measures (i.e., hemoglobin A1c, low-density lipoprotein [LDL], serum sodium) rather than clinically relevant outcomes (e.g., mortality, quality of life, strokes)?
- Does the published (or unpublished) literature contain conflicting evidence about efficacy? Is there suggestion of selective publication, or selective sharing of only more favorable studies by those advocating formulary addition.
- What is the “marginal efficacy”—efficacy above and beyond other therapeutic alternatives?
- Do the efficacy studies use proprietary or manufacturer-developed scales that may bias the findings to give favorable results (e.g., specialized, manufacturer-developed quality of life instruments targeted to be responsive to the effects of a particular drug)?

**C. Safety**

***What safety issues need to be considered?***

- Is there a potential for look-alike, sound-alike name errors raised by or reported for this drug?
- Are there safety issues surrounding the administration or preparation requirements?
- What is the adequacy of the experience with the drug? What are the number and types of patients studied? How long has the drug been used to assure there is a demonstrated safety track record (since many adverse effects only appear after 5–10 years of use)?
- Are there suggestions of early warning signals (either in the literature, unpublished studies or reports, or theoretical concerns based on class effects) of potential safety concerns (e.g., reports of hepatotoxicity, nephrotoxicity, or drug–drug interactions, QT prolongation) that may be a red flag, cautioning against moving too quickly to approve the drug?
- What patient monitoring or other special precautions (e.g., pregnant women, renal insufficiency, government-mandated risk evaluation and mitigation strategies [REMS in US]), are needed or required to use the drug safely? How difficult will it be for practitioners to comply with needed monitoring, and how likely are they to perform adequately?
- How strong is the evidence of this drug's safety compared to other drugs in its class, or other drugs for the same indication currently on the market? What are the anticipated types of adverse events? How do the frequency, severity, preventability, and ameliorability of these adverse events compare across alternative drugs for this indication?

**D. Misuse impact potential**

***If placed on the formulary, what is the potential for misuse or overuse?***

- Is the drug subject to intensive marketing to either consumers or prescribers for questionable and/or off-label indications that may lead to excessive or inappropriate use?
- Is there evidence or worry that the drug will be subject to excessive or unrealistic patient demand and expectation? Are there concerns that advertising, including direct-to-consumer in countries where this exists, will play a role in patient demand? Are industry-funded patient advocacy groups aggressively lobbying for the drug, possibly creating pressures for premature or overuse?
- Is there uncertainty or difficulty in accurately diagnosing the condition that is the indication for this drug, leading to potential overuse or inappropriate use of the drug?
- Are there concerns for widespread “off-label” usage?
- Might the expansion of indications to new manufacturer-promoted syndromes play a role in this drug's usage and potential for overuse?
- Is there experience (in our institution or published literature) with similar drugs and situations suggesting there may be overuse of this agent?
- Is there evidence that any of the active ingredients in the drug is addictive or habit forming?

**E. Cost Implications**

***Can we justify the cost of this drug?***

- How much will it cost? Are there other relevant costs such as additional preparation, storage, administration, monitoring, or other downstream costs beyond simple acquisition costs?
- What is the cost and burden of additional monitoring requirements in safely using this drug?
- What are the comparative costs of other alternatives (e.g., are generics available?)
- Will a competitor/comparable drug soon become available generically?
- If there is an added cost associated with using this drug, is there a significant clinical benefit that justifies the added expense?
- What other pricing issues (rebate deals, market share or exclusivity requirements, some of which may not be transparent) may impact purchasing this drug for our institution or patients? Will the price be raised later once we switch over to this drug (“bait and switch” pricing tactics)?
- What are re-imbursement cost ramifications? What costs will be covered by private or public insurers versus what costs will be borne by the institution or patients (as co- or full pay)?
- What costs are involved in switching patients currently on another drug that we may be substituting this medication for (additional visits, monitoring)?
- Is pill-splitting a possibility for cost savings? Is it easy, safe, desirable?
- How do the acquisition and above additional costs compare to evidence of cost savings (reduction in admissions, other expenditures)?

**F. Decision-making information, calculations, timing, and process**

***What is the strength and quality of evidence and information available to the Committee?***

- What is the source (i.e., from pharmaceutical sales representative versus independent review), completeness, timeliness, and quality of the information the Committee has available to make a decision at this time?
- Has an independent drug monograph review been prepared for the Committee (e.g., by a pharmacist or drug information service)? If yes: Are the monograph and other information upon which decisions are being made adequate, or are there unanswered questions (such as those raised in this document) that require additional information?
- Are there reviews by other formulary or guideline committees or international drug bulletins whose judgments and decisions can also help inform our discussion and decision?
- Are there outstanding questions that may be answered by additional information (e.g., pending research trials) that may warrant deferring a decision?

***What is the status and quality of the review process and use at our institution?***

- Has the drug previously been reviewed by our formulary Committee? If yes what were the issues raised in prior review, discussion, and decision? Was the process a fair and high quality group decision?
- Have there been significant numbers of non-formulary requests for this drug at our institution or plan? If yes, what are utilization and safety experiences and issues surrounding its non-formulary use? What are the pros and cons of keeping non-formulary status for now?
- Have the requisite subcommittees and key and knowledgeable specialists been consulted, how have they weighed in on the decision?
- Has there been undue influence or bias impacting the decision-making process? Have all conflicts of interest (financial, research funding) been disclosed related to the requester, committee members, or involved with evaluating this drug's formulary status (e.g., desire to please a high income-generating clinician)?
- What is desirability of approval now versus delaying approval pending additional information?
- Which clinicians should be permitted to use this drug and in what clinical venue?
- Should there be restrictions (e.g., clinical prior approval or other mechanisms) placed on this medication (based on indication, safety, clinical, or cost outcomes)? If so, what should they be and how easily can they be operationalized and made to work effectively minimizing administrative burdens?
- Are there guidelines and/or electronic clinical decision support alerts that could help ensure safe and appropriate use of this medication; how can they best be operationalized?

The tool is organized around the following six broad questions: (A) Evidence of need: Is there compelling evidence of a need to add this drug to our formulary? (B) Efficacy: What is the strength and quality of evidence to support claims for this drug? (C) Safety: What safety issues need to be considered? (D) Misuse impact potential: If placed on the formulary, what is the potential for misuse or overuse? (E) Cost Issues: Can we justify the cost of this drug? (F) Decision-making information, calculations, timing and process: What is the quality and completeness of evidence, and deliberations of committee?

## Effective Use of the FLIP Tool

To most effectively use this tool, a number of caveats should be kept in mind. First, given the rich diversity of formularies and formulary committees, it would be difficult to standardize a one-size-fits-all tool—one equally applicable to all national and local, inpatient and outpatient settings, public sector and private sector, more restrictive and less controlled formulary-based drug management environments and plans. Second, it is unrealistic to expect that every question would be addressed for every drug at every meeting, certainly during the limited discussion time typically available at formulary committee meetings. Thus, we present a comprehensive list of questions that may be more or less applicable depending on the setting and drug. To save time during the committee meetings, we anticipate that the investigation of many of the checklist questions might be done before rather than during the meeting perhaps by a clinical pharmacist preparing the drug monograph.

Formulary committees often have limited time to make decisions, but an even larger constraint is the paucity of evidence, or at least high quality evidence, to answer with confidence many of the questions the checklist poses. This limitation is particularly true for comparative effectiveness assessments, a shortcoming that has recently been prominently spotlighted but is one that has long challenged thoughtful formulary decision makers [26],[27]. While acknowledging this limitation in applying the tool, we also view it as a strength, in that the unanswerable questions can serve to highlight evidence gaps and force the committee to prioritize the key questions for drugs being considered, underscoring the need to proceed with caution when crucial evidence is lacking.

With these caveats in mind, we suggest the FLIP formulary drug evaluation tool could be used in a number of ways, depending on the type and scope of the formulary committee as well as the time and resources available. In many settings there is an individual (e.g., a clinical pharmacist) or group charged with preparing a dossier of material for the committee prior to the meeting to consider a drug. The FLIP tool could be used to structure this dossier, organizing the material by topic and identifying gaps in the evidence and unanswered questions. Alternatively, the tool could be used to organize and guide discussion during the committee meeting. It could also be used to educate new members of the committee about the kinds of questions that need to be raised when evaluating drugs for formulary placement. Finally it could be used for quality assessment and improvement of the formulary committee processes itself, for example by recording or reviewing formulary meetings and then using the tool to determine how many of the key questions were raised, how well they were addressed, to examine how effectively the formulary processes fulfilled its role in overseeing critical, evidence-based drug policies.

## Discussion

In a recent review of the international literature of the formulary decision-making process at the hospital level, Ouachi et al. concluded that there is a lack of standardized procedures or methods for decision making [5]. Given this gap and the important role of the local and national formularies in promoting rational prescribing and aiding prescribers, institutions, and insurers in evaluating drugs, particularly new drugs, there is a need to explicitly and systematically pose critical questions that often fail to be asked or addressed in claims for new drugs [9],[13].

The tool frames questions in such a way as to look for evidence of benefit and safety before placing drugs on the formulary, shifting the burden of proof onto those who would advocate placing a drug on the formulary, rather than the default assumption that, unless there is a reason to the contrary, all licensed drugs should be included. This assumption embodies the precepts of essential medicines as well principles of conservative and cost-effective prescribing [28],[29]. The questions based on these principles assume that, lacking evidence of superior safety, efficacy, or other comparative benefit, we ought not to be exposing patients or promoting and paying for expensive new drugs whose risks are poorly understood [19],[29].

Some might object to this shifting of the burden of proof onto proponents of adding drugs, arguing that it represents a bias against newer drugs and their manufacturers. However, it seems appropriate to shift this responsibility to those who develop and market new drugs because they are ethically and legally charged with producing evidence of their product's safety and effectiveness. Pharmaceutical manufacturers increasingly recognize this responsibility to more rigorously address these questions, and thus should welcome more clearly defined and standardized approaches.

We acknowledge that underutilization of appropriate medications is also a problem [26]. However, many underutilized drugs are generic drugs that have already found a place on the formulary. Further, formularies are primarily responsible for ensuring that needed drugs are made available, not necessarily that their use is promoted. Rather the tool is designed to support a critical function of the formulary—to serve as counterweight—balancing other forces that tend to promote more liberal use of drugs (e.g., advertising, patient demands, time pressure, inability of clinicians to have the time and expertise to critically review claims).

The tool has not been systematically evaluated to determine the impact of its deployment on decision-making processes, committee decisions, or clinical outcomes. While some data exist demonstrating that effective formularies can restrain costs [30],[31], there are few high quality studies demonstrating the ways formularies actually improve clinical outcomes—studies sorely needed given how many lives and potentially adverse outcomes (either caused or prevented) are at stake. Box 2 lists key questions that such studies, particularly as they pertain to this tool, will need to address. The Academy of Managed Care Pharmacy has adopted an earlier version of our checklist as an appendix to version 3.0 of the AMCP Format for Formulary Submissions—the standard template that managed care organizations use to request information from manufacturers about products being considered by formulary committees. AMCP's adoption of the tool suggests that one influential US organization finds the tool to be potentially useful for its members, a limited but influential subset of the target audience for this tool [32].

**Box 2. Evaluating and Sharpening the Formulary Decision-Making Tool: Questions to Further Validate and Evaluate This Tool**

**Usability**

Are the questions clear, reliably interpreted by members?

How easily can checklist be deployed by the Committee?

Is it feasible to address these questions (versus insufficient information, time)?

**Relevance**

Are these the high priority questions for the formulary committee (in general, for that particular committee)?

Are there important domains/questions overlooked?

Does it include unnecessary or less relevant questions?

**Impacts**

How does the tool impact decision-making process and outcomes?

In what ways does it help guide meeting discussions?

What is its impact on committee decisions, patient outcomes, costs?

**Educational value and user satisfaction**

Is it serving a useful educational purpose (orientating new members, others)?

Do committee members like it and find it helpful?

**Broader (more indirect and speculative) impacts**

Does the tool enhance committee transparency, credibility, status/recruitment of members?

Does it promote higher quality of monographs, materials prepared/distributed?

Will it lead to more standardized drug lists across settings?

Does it promote better support for formulary processes; lead to more regional/centralized formulary committees?

Impacts on industry—does it help create clearer expectations, raise bar for stimulating better information, better studies, or even better drugs?

**Adverse effects**

Is use of the tool too costly, requiring excessive staff time to prepare/address?

Does it lead to keeping drugs off formulary that are later proven highly valuable (i.e., does it make the formulary overly restrictive)?

Might its rigor and promotion of a stricter formulary antagonize clinicians, negatively impacting buy-in for formulary/decisions?

Our tool was found to be useful in two US public hospital formularies. While not representative of all settings and uses, one hospital employs a formulary that is more common in the US (overseeing only the inpatient formulary), while the other operates more on a “single payer” fixed budget and oversees both inpatient and outpatient drugs. Thus its applicability appears to be reasonably broad. Here, we offer the tool for a broader audience to use and test. We hope that it will prove useful in improving the quality of formulary decision making and stimulating debate related to critical questions that need to be asked, highlighting essential data needed to more safely prescribe drugs. As a guide to posing critical questions related to drugs being adopted, it represents a starting point, for both local formulary committees and national policymakers to use in their evaluations of new drugs for formulary inclusion as well as to further evaluate, test, and refine.
